# Effects of Omegaven®, EPA, DHA and oxaliplatin on oesophageal adenocarcinoma cell lines growth, cytokine and cell signal biomarkers expression

**DOI:** 10.1186/s12944-018-0664-1

**Published:** 2018-01-30

**Authors:** Amar M. Eltweri, Lynne M. Howells, Anne L. Thomas, Ashley R. Dennison, David J. Bowrey

**Affiliations:** 1 Department of Surgery, University Hospitals of Leicester NHS Trust, Leicester Royal Infirmary, Leicester, LE1 5WW UK; 2 Department of Cancer Studies, University of Leicester, Leicester, LE2 7LX UK

## Abstract

**Background:**

There is limited evidence assessing the effects of omega-3 polyunsaturated fatty acids (PUFAs) on oesophageal adenocarcinoma, both in vitro and in vivo. We evaluated the effects of the omega-3 PUFA and oxaliplatin on OE33 and OE19 cells.

**Method:**

The two oesophageal cells were treated with Omegaven® (fish oil emulsion), EPA, DHA and oxaliplatin and incubated for up to 144 h.

**Results:**

The following inhibitory effects were observed on OE33 cells: EPA reduced cell growth by 39% (*p* = 0.001), DHA by 59% (*p* < 0.000) and Oxaliplatin by 77% (*p* < 0.000). For OE19 cells, the EPA reduced growth by 1% (*p* = 0.992), DHA by 26% (*p* = 0.019) and oxaliplatin by 76% (*p* < 0.000). For both cells, Omegaven**®** resulted in reduced cell growth at intermediate concentrations (20-40 μM) and increased cell growth at low (10 μM) and high (50 μM) concentrations. DHA, Omegaven**®** and oxaliplatin were associated with significant downregulation of VEGF and p53 protein, and upregulation of p21 protein. DHA, Omegaven**®** and Oxaliplatin also led to significant downregulation of the total ERK1/2 and Akt proteins.

**Conclusion:**

DHA, Omegaven® and oxaliplatin were associated with downregulation of p53 and VEGF in both cells. Of the PUFAs studied, DHA alone or in combination (Omegaven®) had greater in vitro anti-cancer effects than EPA alone.

**Electronic supplementary material:**

The online version of this article (10.1186/s12944-018-0664-1) contains supplementary material, which is available to authorized users.

## Background

There is an increasing interest in natural therapies with anti-inflammatory effects that exhibit anticancer benefit, including omega-3 PUFAs. The main omega-3 PUFA are eicosapentaenoic acid (EPA) and docosahexaenoic acid (DHA) which have anti-inflammatory effects [1–3]. Only a small amount of these PUFAs can be synthesized in the human body, approximately 2–10% of α-linolenic acid is converted to EPA and DHA. Hence, it is mainly dependent on external sources. The use of EPA and DHA has been extensively investigated, with proven in vitro anticancer effects in gastrointestinal cancers such as colorectal and pancreatic adenocarcinoma. However, there is limited evidence in oesophageal cancer as highlighted by the author’s published review [4].

Several factors in the cancer microenvironment influence the carcinogenesis process, including the delivery of bioactive molecules, such as cytokines and growth factors that are responsible for increased cell proliferation, inhibition of apoptosis and induction of angiogenesis [5]. In many patients, oesophagogastric cancer is considered to arise as a consequence of chronic inflammation [6]. This link is implicated for the development of gastric cancer as a result of *Helicobacter pylori* (*H. pylori*) related chronic gastritis and atrophy [7]. It is established that *H. pylori* infection causes the induction of various pro-inflammatory cytokines such as TNF-α, Interleukin (IL) -1 and IL-6 [7]. For the oesophagus cancer, the link between inflammation and cancer is strongest for adenocarcinoma as a result of chronic reflux associated inflammation [6].

Wu et al., treated gastric cancer cell lines with EPA and DHA, and found inhibited macrophage activated cell migration by down regulation of the matrix metalloproteinase 10 gene, and subsequent down regulation of extracellular signal-related kinase (ERK) [8]. Slagsvold et al. showed that DHA (75 μM) had significant anticancer effects on colon cancer cell lines, causing cell cycle arrest through upregulation of p21 protein and downregulation of survivin and livin (inhibitors of apoptosis) [9].

In this exploratory study, we evaluated the effect of the four single treatments (EPA, DHA, Omegaven® (fish oil emulsion) and oxaliplatin) on OE33 and OE19 cell growth and expression of the following cytokines: IL-6, TNF-α and VEGF in the cell culture supernatant. In addition, we also evaluated expression of the following proteins p53, p21, Akt, ERK1/2 in the cell lysate.

## Methods

The two oesophageal cancer cell lines used were OE19 and OE33. OE19 is a human oesophageal cancer cell line derived from a 72 year old white male patient with moderately differentiated UICC stage 3 adenocarcinoma. The OE33 cancer cell line is derived from a 73 year old white female with UICC stage 2A lower oesophageal adenocarcinoma arising in a background of known Barrett’s metaplasia. These cell lines were purchased from Public Health England cell collection (The European Collection of Authenticated Cell Cultures).

### Maintenance of cell lines

Cell lines were cultured as a monolayer at 37 °C and 5% CO_2_. Both cell lines were cultured in RPMI 1640 medium (Sigma-Aldrich, UK) supplemented with 2 mM Glutamine and 10% foetal bovine serum (FBS).

### Cell passaging

Cell lines were passaged no more than 15 times following resuscitation from liquid nitrogen, to reduce the risk of phenotypic alterations. Passaging was undertaken once cells had reached approximately 80% confluence as follows: Cells were washed with 10 mL pre-warmed (37 °C) PBS once, followed by addition of 5 mL of 1X trypsin for 5 min at 37 °C for cell detachment. The trypsinisation process was halted following addition of an equivalent volume of RPMI media containing 10% FBS. Cells were pelleted at 400 x g, resuspended in fresh medium containing 10% FBS, and aliquoted appropriately into cell culture flasks as per experimental requirements.

### Treatments and solvents

The treatments tested were EPA, DHA, Oxaliplatin (all from Sigma-Aldrich, UK), and Omegaven® (Fresenius Kabi, Germany). EPA and DHA stocks were prepared as 50 mM stocks dissolved in DMSO and oxaliplatin was prepared as a 50 mM stock dissolved in 5% dextrose. All treatments including the vehicle control, received equivalent volumes of DMSO or 5% dextrose. Omegaven® is a 10% fish oil lipid emulsion containing 1.25 to 2.82 g/100 ml EPA and 1.44 to 3.09 g/100 ml DHA as per the Omegaven® summary of product characteristics. The rationale for selecting Omegaven® was that it was commercially available, the omegaven® emulsion was also investigated over the same time period in a pilot clinical trial in patients with advanced oesophago-gastric cancer and the intention was to mirror the in vitro laboratory work with the clinical trial.

### EPA, DHA, Omegaven*®* and Oxaliplatin treatments

OE33 and OE19 cell lines were grown in RPMI 1640 + 2 mM Glutamine + 10% foetal bovine serum (FBS) medium for 24 h, then the media was removed and replaced with medium containing 10 μM, 20 μM, 30 μM, 40 μM and 50 μM of EPA, DHA and Oxaliplatin treatment and in order to equate the Omegaven® emulsion mixture to treatment concentrations using the single agents, the emulsion was diluted in RPMI medium + 10% FCS via serial dilution to make treatments of approximately 10 μM, 20 μM, 30 μM, 40 μM and 50 μM of EPA and DHA. The cell lines were incubated for 72, 96, 120 or 144 h to determine the anti-proliferative effects. The cell culture supernatant was collected at 72 and 144 h and stored at − 80 °C for cytokine analysis. The cells were then harvested and counted.

### Cell proliferation assays

Cell proliferation was undertaken using a Z2 particle size analyser (Beckman Coulter, UK) to count raw cell numbers; this was performed in both cell lines for comparison in triplicate. OE19 and OE33 cell lines were seeded into 24-well plates at a density of 2 X 10^3^ cells/well in 1 mL of RPMI 1640 medium. Cells were incubated for 24 h, and then the media was replaced with media containing the relevant treatments. Cells were then incubated for 72, 96, 120 and 144 h before counting; Each well was washed × 1 with 1 mL PBS and 0.5 mL of 1X trypsin added to each well, which was neutralised with 0.5 mL RPMI + 10% FBS once cells had detached. The well contents were transferred to coulter count cups containing 9 mL of isoton solution (Beckman Coulter, UK) and cells were counted using the Z2 particle analyser.

### Cytokine and cell signalling biomarkers analysis

#### Cell culture supernatant collection

Cell culture media supernatants were collected at 72 and 144 h after incubation with treatment as described above, and stored at − 80 °C for analysis of IL-6, TNF-α, VEGF using an ELISA assay.

#### Cell lysate preparation

The cell lines (OE19 and OE33) were seeded at 1.5 X 10^5^ cells per 75 cm flask and allowed to adhere overnight. Cells were then treated with EPA, DHA, Omegaven® or Oxaliplatin at concentrations of 10 μM and 50 μM and incubated for 72 and 96 h.

After 72 and 96 h, all flasks were removed from the incubator and placed on ice. Cells were gently scraped using a sterile cell scraper, media containing cells was collected and cells pelleted (400 x g, 4 °C, 3 min). The supernatant was discarded, and the remaining cell pellet washed with 1 mL PBS. Cells were again pelleted, the PBS carefully aspirated and 100 μL of Roche Complete Lysis M (Roche Ltd., UK) cell lysis buffer added to the cell pellet. The pellet was mixed by vigorous pipetting and the cells lysed on ice for 10 min. Cell debris was pelleted and the supernatant stored at − 80 °C for later analysis of total Akt, Erk1/2, p53 and p21 proteins.

### Statistical analysis

The data was not normally distributed hence all data were transformed to log 10. In order to identify whether there was any significant effect of each treatment, a two tailed t test was used to compare cell counts and all cytokine expression compared to the control.

## Results

### Effects of omega-3 PUFAs and oxaliplatin single treatment on cell proliferation

Growth inhibition of both cell lines was observed following treatment with EPA, DHA and oxaliplatin. The growth inhibition occurred in a time-, dose- and cell line dependent manner, with OE33 cells exhibiting more sensitivity to treatment than OE19 cells.

The following inhibitory effects were observed on OE33 cells: Compared to control, EPA reduced cell growth by 39% (*p* = 0.001), DHA reduced cell growth by 59% (*p* < 0.000) and Oxaliplatin reduced cell growth by 77% (*p* < 0.000). For OE19 cells, the EPA reduced growth by 1% (*p* = 0.992), DHA reduced growth by 26% (*p* = 0.019) and oxaliplatin reduced cell growth by 76% (*p* < 0.000). For both cell lines, the effects of Omegaven on cell growth did not follow a linear pattern, but more a “U-shaped” curve, with increased cell growth at low (10 μM) and high (50 μM) concentrations, and decreased cell growth at intermediate (20–40 μM) concentrations, see Additional file 1: Figure 1 a-d.

### Effects of omega-3 PUFAs and oxaliplatin single treatment on cytokine expression

**Fig. 1:**
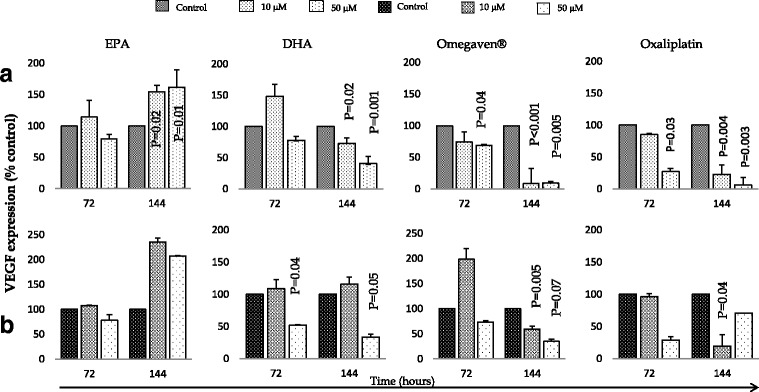
Effects of EPA, DHA, Omegaven® and oxaliplatin treatments over time on VEGF expression in (**a**) OE33 and (**b**) OE19 cells. Analysis was made using ELISA assays in duplicate. The graphs represent the mean and standard deviation (SD) of VEGF concentration (pg/mL) expressed as a percentage of control. Statistical analyses was by two tailed student’s t test of log 10 transformed data, and a *P* value of <0.05 considered significant

In a similar pattern to the cell growth inhibition, prolonged DHA, Omegaven**®** and oxaliplatin treatment was associated with significant down regulation of VEGF expression compared to control. A similar concentration of EPA was associated with an upregulation effect (Fig. 1). Treatment effects on IL-6 and TNF-α expression was also assessed and showed treatment dose and time dependent effect on cytokine expression. Data are presented in Table 1.

**Table 1:** Effects of EPA, DHA, Omegaven® and oxaliplatin treatments over time on OE33 and OE19 cell line expression of IL-6 and TNF alpha

			OE33 cell lines					OE19 cell lines				
			Control	10 μM	Sign.	50 μM	Sign.	Control	10 μM	Sign.	50 μM	Sign.
IL-6	EPA	72 h	3.7 ± 0.33	4.6 ± 0.37	*P = 0.12*	4.3 ± 0.02	*P = 0.18*	3.8 ± 0.35	3.6 ± 0.53	*P = 0.68*	2.9 ± 0.13	*P = 0.06*
		144 h	3.0 ± 0.38	3.6 ± 0.22	*P = 0.18*	5.1 ± 0.17	***P = 0.03***	2.7 ± 0.20	3.2 ± 0.26	*P = 0.13*	3.6 ± 0.06	***P = 0.03***
	DHA	72 h	4.5 ± 0.37	4.8 ± 0.51	*P = 0.60*	4.5 ± 0.11	*P = 0.87*	4.1 ± 0.35	3.7 ± 0.32	*P = 0.43*	3.7 ± 0.13	*P = 0.32*
		144 h	5.6 ± 0.87	6.2 ± 0.02	*P = 0.05*	5.1 ± 0.38	*P = 0.29*	3.9 ± 0.45	4.5 ± 0.06	*P = 0.21*	4.3 ± 0.23	*P = 0.37*
	Omegaven**®**	72 h	4.7 ± 0.20	5.6 ± 0.18	***P = 0.03***	5.6 ± 0.24	***P = 0.04***	3.8 ± 0.05	4.0 ± 0.41	*P = 0.60*	4.7 ± 0.49	*P = 0.09*
		144 h	5.4 ± 0.15	5.2 ± 0.27	*P = 0.32*	4.9 ± 0.30	*P = 0.13*	3.1 ± 0.14	4.0 ± 0.27	*P = 0.05*	4.7 ± 0.07	***P = 0.007***
	Oxaliplatin	72 h	4.9 ± 0.11	5.7 ± 0.15	***P = 0.01***	4.8 ± 0.11	*P = 0.18*	4.0 ± 0.15	4.5 ± 0.44	*P = 0.30*	4.1 ± 0.73	*P = 0.97*
		144 h	4.4 ± 0.00	4.1 ± 0.39	*P = 0.46*	4.7 ± 0.01	*P = 0.08*	3.7 ± 0.54	4.7 ± 0.03	*P = 0.15*	5.8 ± 0.66	*P = 0.07*
TNF alpha	EPA	72 h	2.6 ± 0.55	3.3 ± 0.45	*P = 0.29*	2.7 ± 0.27	*P = 0.82*	2.3 ± 0.12	2.2 ± 0.31	*P = 0.78*	2.2 ± 0.13	*P = 0.42*
		144 h	2.1 ± 0.40	2.5 ± 0.05	*P = 0.28*	3.6 ± 0.23	*P = 0.06*	1.6 ± 0.14	2.1 ± 0.24	*P = 0.14*	2.5 ± 0.13	***P = 0.02***
	DHA	72 h	3.3 ± 0.12	3.9 ± 0.38	*P = 0.85*	3.0 ± 0.49	*P = 0.42*	2.2 ± 0.06	2.3 ± 0.12	*P = 0.40*	2.7 ± 0.11	***P = 0.03***
		144 h	3.8 ± 0.22	4.4 ± 0.13	*P = 0.08*	3.6 ± 0.20	*P = 0.48*	2.3 ± 0.13	2.8 ± 0.00	***P = 0.04***	2.9 ± 0.09	***P = 0.04***
	Omegaven**®**	72 h	3.2 ± 0.05	3.6 ± 0.21	*P = 0.06*	3.7 ± 0.02	***P = 0.008***	2.2 ± 0.08	2.5 ± 0.20	*P = 0.20*	3.3 ± 0.39	***P = 0.04***
		144 h	3.6 ± 0.05	3.3 ± 0.33	*P = 0.34*	2.7 ± 0.30	*P = 0.05*	2.2 ± 0.00	2.6 ± 0.28	*P = 0.17*	3.3 ± 0.03	***P < 0.001***
	Oxaliplatin	72 h	3.3 ± 0.08	4.1 ± 0.20	*P = 0.03*	3.3 ± 0.06	*P = 0.99*	2.5 ± 0.12	2.8 ± 0.20	*P = 0.23*	2.9 ± 0.39	*P = 0.40*
		144 h	2.6 ± 0.00	2.6 ± 0.01	*P = 0.03*	2.9 ± 0.19	*P = 0.11*	2.3 ± 0.34	3.1 ± 0.09	*P = 0.14*	4.1 ± 0.88	*P = 0.10*

Data presented as mean ± SD (pg/ml), two tailed student t test was used to identify any significance of log 10 transformed data, *P* < 0.05 considered significant. Bold text indicate significant upregulation compared to control

### Effects of omega-3 PUFAs and oxaliplatin treatment on cell signal markers expression

**Fig. 2:**
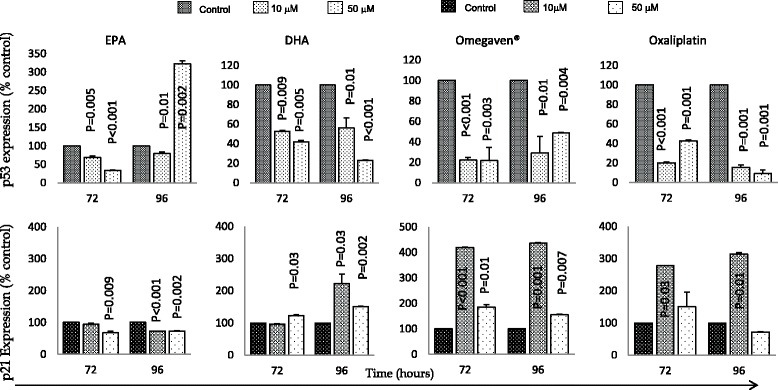
Effects of EPA, DHA, Omegaven® and oxaliplatin treatments over time on p53 (top panel) and p21 (bottom panel) expression in OE33 cells. Analysis was made using ELISA assays in duplicate. The graphs represent the mean and standard deviation (SD) of p53 and p21 proteins (percentage of control). Using two tailed student’s t test of log 10 transformed data and *P* value of <0.05 considered significant

**Fig. 3:**
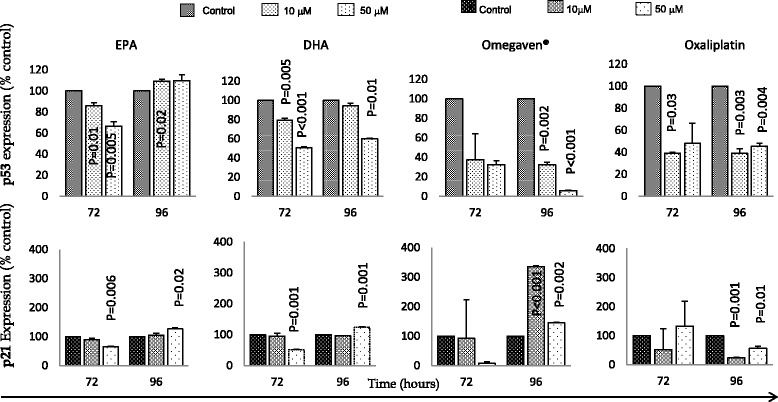
Effects of EPA, DHA, Omegaven® and oxaliplatin treatments over time on p53 (top panel) and p21 (bottom panel) expression in OE19 cells. Analysis was made using ELISA assays in duplicate. The graphs represent the mean and standard deviation (SD) of p53 and p21 proteins (percentage of control). Using two tailed student’s t test of log 10 transformed data and *P* value of <0.05 considered significant

To determine if the anti-proliferative effects observed with the single treatments was associated with changes in cell signalling markers; we assessed the expression of the total p53, p21 proteins as well as Akt and ERK-1/2 proteins. Interestingly, despite both cells being p53 mutant, DHA, Omegaven and Oxaliplatin treatment were associated with significant downregulation of p53 protein expression and upregulation of p21 proteins when compared to the control, whereas, for the EPA time and concentration dependent (Figs. 2 and 3).

**Fig. 4:**
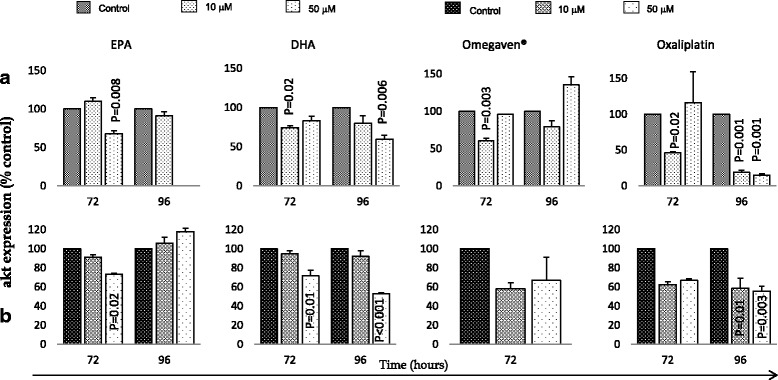
Effects of EPA, DHA, Omegaven® and oxaliplatin treatments over time on akt expression in OE33 (**a**) and OE19 (**b**) cells. Analysis was made using ELISA assays in duplicate. The graphs represent the mean and standard deviation (SD) of akt (percentage of control). Using two tailed student’s t test of log 10 transformed data and *P* value of <0.05 considered significant. The OE19 cell lines akt expression data (Control) for omegaven® treatment was not read during ELISA assay (technical issues), hence not presented

**Fig. 5:**
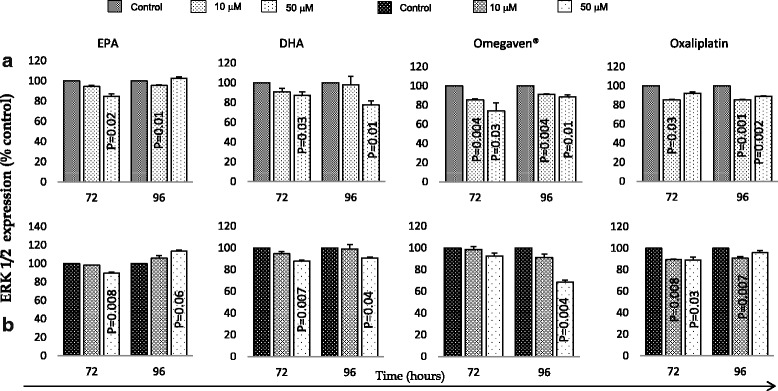
Effects of EPA, DHA, Omegaven® and oxaliplatin treatments over time on ERK 1/2 expression in OE33 (**a**) and OE19 (**b**) cells. Analysis was made using ELISA assays in duplicate. The graphs represent the mean and standard deviation (SD) ERK 1/2 (percentage of control). Using two tailed student’s t test of log 10 transformed data and *P* value of <0.05 considered significant

DHA and oxaliplatin (50 μM) significantly downregulated Akt expression in both cell lines after 96 h (Fig. 4). All treatments were associated with similar downregulation effect on ERK 1/2 expression in both cells (Fig. 5).

## Discussion

It has been reported in four previous studies on gastric adenocarcinoma cell lines that omega-3 PUFAs (mainly EPA and DHA) present in fish oil are associated with anti-proliferative effects [8, 10–12], the cellular mechanisms accounting for these effects included modulation of p53 dependent [13] and independent pathways, suppression of nuclear factor Kappa-light chain enhancer of activated B cells (NF-kB) [14], down regulation of Bcl-2 expression [12] and inhibition of cyclo-oxygenase-2 (Cox-2) [14], but the literature lacks similar evidence in oesophageal adenocarcinoma cell lines [4].

We have shown for the first time that EPA, DHA, and oxalipatin have anti-proliferative effects on OE33 and OE19 cell lines. There was a more pronounced dose response effect with DHA and oxaliplatin than with EPA. Oxaliplatin as a single treatment or coupled with EPA has been previously investigated in colorectal cancer cell lines [15, 16]. These studies demonstrated that the addition of omega-3 PUFAs enhanced the oxaliplatin cytotoxic effects and reduced cell growth [15, 16]. In this study, oxaliplatin was used as a single treatment only and was associated with significant reduction in cell growth from as low as 10 μM. The combination of oxaliplatin and omega-3 PUFAs was not explored in the current series of experiments, but this remains the potential next avenue of research.

This study differs from previous studies that investigated omega-3 PUFAs in that we used EPA and DHA as a single treatment and in combination in the form of a fish oil emulsion (Omegaven®) in oesophageal adenocarcinoma cell lines for the first time. At low (10 μM) and high (50 μM) concentrations of Omegaven®, treatment resulted in a significant increase in cell number for both OE19 and OE33 cell lines, this effect was lost at intermediate concentrations of 20 μM and 30 μM as shown in the Additional file 1: Figure 1 a-d. This has been previously investigated in colorectal cancer cells and the findings were similar to our study in that higher concentrations of Omegaven® emulsion (0.72–1.44 mL/L equivalent to 50–100 μM of EPA) also led to a pro-proliferative effect [17]. This type of concentration dependent effect has also been reported previously in breast cancer cell lines using another dietary agent (Genistein), with the authors reporting increased cell proliferation at lower concentrations (1 μM) and decreased proliferation at higher (cytotoxic) genistein concentration (25 μM) [18].

Granci et al., investigated the effects of Omegaven® (EPA and DHA equivalent of 24 μM and 20.5 μM respectively) in combination with oxaliplatin in colorectal cancer HT-29 cells, with this combination associated with further significant reduction of cell viability when compared to oxaliplatin alone [19].

Omegaven® emulsion has various other substances such as preservatives, stabilisers and also other fatty acids including sodium oleate (monounsaturated fatty acid). The latter might theoretically have altered the growth characteristics of the oesophageal cancer cell lines as it had previously reported to increase breast cell line proliferation and decrease apoptosis [20, 21]. In addition, it was very challenging to calculate the accurate equivalent concentrations of EPA and DHA, as each 100 mL bottle of Omegaven® merely specified a concentration range; 1.25–2.82 g of EPA and 1.44–3.09 g of DHA due to the natural character of the product.

In colonic cancer cells, both EPA and DHA (10–30 μM) were associated with inhibition of VEGF expression [22]. In vivo, mice injected with colorectal cancer cells and fed EPA and/or DHA were associated with a reduction in tumour size and reduced expression of VEGF [22]. Higher baseline serum VEGF levels have been shown to be associated with poor survival [23], and promotion of metastasis [24].

In the current series of experiments, the most profound effects on cytokine production were on VEGF. The treatment of OE33 cells with DHA, Omegaven® and oxaliplatin in this experiment, showed significant decreases in VEGF expression after prolonged treatment of 144 h (Fig. 1). The changes in OE19 cells were less marked than those seen on OE33 cells. Although both cell lines are derived from tumour tissue from patients with oesophageal adenocarcinoma, the OE33 cell lines arose in the setting of extensive Barrett’s oesophagus. These phenotypic differences may explain some of the differences in cytokine expression under exogenous influences.

The effect of treatment on IL-6 and TNF-α expression by both OE33 and OE19 cells (unstimulated) was examined and we found that EPA treatment was associated with a significant increase in IL-6 and TNF-α expression as early as 96 h of treatment in OE33 cells and 120 h in OE19 cells. DHA, Omegaven® and oxaliplatin led to dose- and time dependent biphasic changes in OE33 cells. This sort of inconsistent low dose vs high dose effect appears to be a common finding in response to a variety of dietary agents and has previously been reported by Lavigne et al. in 2008, while investigating the effects of isoflavones (Genistein) in breast cancer cell lines [18]. Cai H et al. demonstrated a low dietary intake of resveratrol is more effective in preventing colorectal cancer development in animal models than higher intake [25].

It had been previously reported that omega-3 PUFAs inhibited the activation of NF-kB, which normally regulates the Interleukin-6 and TNF-α expression [26, 27]. The suppression of NF- kB protein expression in colorectal adenocarcinoma cell lines (CaCo-2) treated with 5 μM of DHA [14] and pancreatic cells treated with higher dose of EPA (100 μM) was reported. [27] The treatments dose used in this experiment were different from the previous studies, and this might explain the different findings reported in this study. These effects appear to be common in response to a variety of dietary agents [18].

The two oesophageal cells used in this study were p53 mutant. However, DHA, Omegaven**®** and oxaliplatin treatments in both cells were associated with significant downregulation of p53 and upregulation of p21 protein, but this was not the case for EPA (Figs. 2 and 3). Kato et al. [28], reported that DHA but not EPA was the primary tumour suppressing omega-3 PUFA, and that it inhibited cancer growth by p53 dependent and independent pathways in colonic cancer [28]. Moreover, all treatments used in this study were associated with downregulation of the total expression of both ERK 1/2 and Akt expression. Taken together, these findings suggest that the effects of Omegaven**®**, EPA, DHA and oxaliplatin on apoptosis was possibly through inhibition of the MAPK cascade and that DHA inhibited growth of both cell lines in a p53 independent manner.

The novelty of this study was that we investigated the effects of EPA, and DHA as single agents and used Omegaven® for the first time in oesophageal adenocarcinoma cell lines. The assessment of various pro-inflammatory cytokines and cell signalling biomarkers forms the initial evidence of the effects of omega-3 PUFAs in oesophageal adenocarcinoma cell lines, forming the basis for future research identifying potential mechanisms of action for cell growth inhibition. Nevertheless, there are limitations to the current studies. All markers were assessed using ELISA without subsequent confirmation by Western blot analysis. Further, the current studies measured only the total protein concentrations as an endpoint read-out but not the phosphorylated (active) forms of these proteins. Future work should assess the effects of treatment on protein phosphorylation for both Akt and ERK 1/2, which would give a more comprehensive indication of which up-stream pathways are directly implicated in regulating PUFA efficacy.

## Conclusion

We have demonstrated that DHA, Omegaven® and oxaliplatin were associated with downregulation of p53 and VEGF in two oesophageal adenocarcinoma cell lines. Of the PUFAs studied, DHA alone or in combination (Omegaven®) had greater in vitro anti-cancer effects than EPA alone. It appeared to exert its anti-cancer effects via multiple different pathways. Further, the magnitude of the anti-proliferative effect observed for DHA was similar to that observed with oxaliplatin in these oesophageal cancer cell lines. We speculate that it could be used as an adjunct to chemotherapy for oesophageal adenocarcinoma to enhance treatment efficacy.

## Additional file

Additional file 1: Figure S1a-d. **Figure S1a**: Effects of EPA treatment on OE19 (A) and OE33 (B) cell line growth at four time points.** Figure S1b**: Effects of DHA treatment on OE19 (A) and OE33 (B) cell growth at four time points. **Figure S1c**: Effects of Oxaliplatin treatment on OE19 (A) and OE33 (B) cell lines growth at four time points. **Figure S1d**: Effects of Omegaven® treatment on OE19 (A) and OE33 (B) cell lines growth at four time points. (DOCX 285 kb)
