# Supplementary material for: Effects of Omegaven®, EPA, DHA and oxaliplatin on oesophageal adenocarcinoma cell lines growth, cytokine and cell signal biomarkers expression
Source: Lipids Health Dis. 2018 Jan 30;17:19. doi: 10.1186/s12944-018-0664-1 (PMC5789622; doi:10.1186/s12944-018-0664-1)
Supplement: Additional file 1: Figure S1a-d. — Figure S1a: Effects of EPA treatment on OE19 (A) and OE33 (B) cell line growth at four time points. Figure S1b: Effects of DHA treatment on OE19 (A) and OE33 (B) cell growth at four time points. Figure S1c: Effects of Oxaliplatin treatment on OE19 (A) and OE33 (B) cell lines growth at four time points. Figure S1d: Effects of Omegaven® treatment on OE19 (A) and OE33 (B) cell lines growth at four time points. (DOCX 285 kb) [file 12944_2018_664_MOESM1_ESM.docx]

**Supplementary figure 1a:** Effects of EPA treatment on OE19 (A) and OE33 (B) cell line growth at four time points

*Lines represent median of the cell count and whisker bars represent interquartile range of three experiments in triplicate, dots represent outliers. Two tailed student’s t test (log transformed data) was used to identify if there was any difference between each EPA concentration and the control (DMSO).*

**Supplementary figure 1b:** Effects of DHA treatment on OE19 (A) and OE33 (B) cell growth at four time points.

*Lines represent median of the cell count and whisker bars represent interquartile range of three experiments in triplicate, dots represent outliers. Two tailed student’s t test (log transformed data) was used to identify if there was any difference between each DHA concentration and the control (DMSO).*

**Supplementary figure 1c:** Effects of Oxaliplatin treatment on OE19 (A) and OE33 (B) cell lines growth at four time points.

B

P=0.01

P=0.014

P=0.013

P=0.011

P=0.001

P=0.001

P<0.0001

P=0.001

P=0.001

P<0.0001

P<0.0001

0

10,000

20,000

30,000

40,000

Cell count

Oxaliplatin

72 hrs

96 hrs

120 hrs

144 hrs

Control

10 µM

20 µM

30 µM

40 µM

50 µM

*Lines represent median of the cell count and whisker bars represent interquartile range of three experiments in triplicate, dots represent outliers. Two tailed student’s t test (log transformed data) was used to identify if there was any difference between each oxaliplatin concentration and the control (5% dextrose).*

**Supplementary figure 1d:** Effects of Omegaven® treatment on OE19 (A) and OE33 (B) cell lines growth at four time points.

*Lines represent median of the cell count and whisker bars represent interquartile range of three experiments in triplicate, dots represent outliers. Two tailed student’s t test (log transformed data) was used to identify if there was any difference between each Omegaven® concentration and the control.*
